# Rare Primary Adrenal Tumor: A Case Report of Teratomas and Literatures Review

**DOI:** 10.3389/fonc.2022.830003

**Published:** 2022-05-09

**Authors:** Xiaomin Wang, Xiaoguang Li, Hongjia Cai, Wei Xiao, Peng Su, Xiang Huang, Xu Luo, Neng Zhang, Ni Fu

**Affiliations:** ^1^ Department of Urology, The Affiliated Hospital of Zunyi Medical University, Zunyi, China; ^2^ Department of Urology, The Second Affiliated Hospital of Zunyi Medical University, Zunyi, China

**Keywords:** teratoma, adrenal gland, imaging and pathological features, case report, laparoscopic resection

## Abstract

Teratomas are very rare, originating from embryonal germ layers. The majority of them are mature, most common in the gonads, and with only 15% out of gonads. In particular, primary adrenal teratomas are extremely rare. The present study reported a case of a young female patient with right adrenal tumor who underwent intermittent pain in the right waist and abdomen and whose CT of adrenal gland showed an 88 mm × 79 mm × 69 mm mass. Besides, her adrenal gland-related hormones are not abnormal. Laparoscopic adrenal tumor resection was performed on her and the histopathological results confirmed that the mass was mature adrenal teratomas. As a newly diagnosed case, strict and regular follow-up is needed, and it is also necessary to detect her AFP and check her adrenal CT in the future. In addition, we have reviewed the literature from 1952 to the present, and a total of 49 cases of adrenal teratoma have been identified and analyzed.

## Introduction

Teratomas are tumor stemming from embryonal germ layers that are mainly composed of pluripotent stem cells. Teratomas are very rare and uncommon, whose incidence is 0.9/100,000 among the population (1); thus, only few cases were published in literature. The majority of teratomas are mature and contain multiple layers of embryonic germ cell layer, with the ectoderm accounting for the majority and the endoderm constituting the least part; therefore, teratomas may contain skin, hair, teeth, brain tissue, nerves, adipose tissue, cartilage, and so forth (2). Based on the original locations of a teratoma, the most common sites of occurrence are the gonads (3), with extragonadal sites including the anterior mediastinum, retroperitoneum, sacrococcygeal region, pineal gland, and suprasellar region (4). Among the retroperitoneal teratomas, primary adrenal teratomas are extremely rare.

According to the differentiation degree of teratomas tissue, teratoma can be divided into two types, namely, mature teratoma and malignant teratoma, which consists of tissue derived from more than one kind of embryonic germ cell layer germ cell layers, including ectoderm, mesoderm, and endoderm (5). Although most teratomas are mature teratomas, which are benign, they have the potential to become malignant and, as they increase in size, can compress surrounding organs and even rupture bleeding and infection. Therefore, once diagnosed, surgery should be performed as soon as possible (6). The surgeon may choose open surgery or laparoscopic surgery, based on the patient’s specific condition and the surgeon’s own experience, and the key to treatment is complete removal of the tumor (7). Although the prognosis after resection of mature teratomas is good with an overall 5-year survival rate of nearly 100% (8), regular post-operative follow-up is necessary because of the risk of malignant regression. Postoperatively, malignant teratomas are prone to recurrence and require adjuvant radiotherapy and chemotherapy with a lifelong follow-up (9). The present study reported a case of a primary right adrenal mature teratoma and aimed to further raise awareness of this rare disease by elucidating the clinical and pathological characteristics of primary adrenal teratoma and by supplementing relevant treatment experience.

## Case Presentation

A 19-year-old female patient presented to the outpatient department of our hospital with the symptoms of intermittent pain in the right waist and abdomen for 4 months; she had no other symptoms. The patient had received no specific treatment prior to this, she follows a healthy daily diet and routine, and no relatives in her family had similar illnesses. After hospitalization, the patient’s temperature, heart rate, breath frequency, blood pressure, and oxygen saturation were normal. A mass was palpated on the upper pole of the right kidney area, about 10 cm × 8 cm in size, with mild percussion pain, but no bulge, no pressure, or percussion pain in both sides of the ureteral region.

Cortisol (COR): 36.5 nmol/L, β-HCG < 0.1 MIU/ml (non-pregnant women 0–5 MIU/ml), AFP: 3.1 ng/ml (<9 ng/ml), CEA: 0.6 μg/L (non-pregnant women <3 μg/L, smoking <5 μg/L), CA19-9: 4 U/ml (<25 U/ml), renin: 1.09 ng/ml, angiotensin I: 1.62 ng/ml, and angiotensin II: 38.77 pg/ml; other indexes were normal and are shown in **Table 1**.

**Table 1:** Detection of adrenal gland related endocrine.

Indexes	Actual value	Range
Fasting glucose	4.2 mmol/L	3.9–6.1 mmol/L
Cortisol (COR)	36.5 nmol/L	
β-HCG	<0.1 MIU/ml	<3.1 MIU/ml
AFP	3.1 ng/ml	<9 ng/ml
CEA	0.6 μg/L	Non-pregnant women<3 μg/L, Smoking<5 μg/L
CA19-9	4 U/ml	<25 U/ml
CA15-3	4 U/ml	<31 U/ml
CA125	7 U/ml	<35 U/ml
Renin	1.09 ng/ml	None
Angiotensin I	1.62 ng/ml	None
Angiotensin II	38.77 pg/ml	None

**Figure 1:**
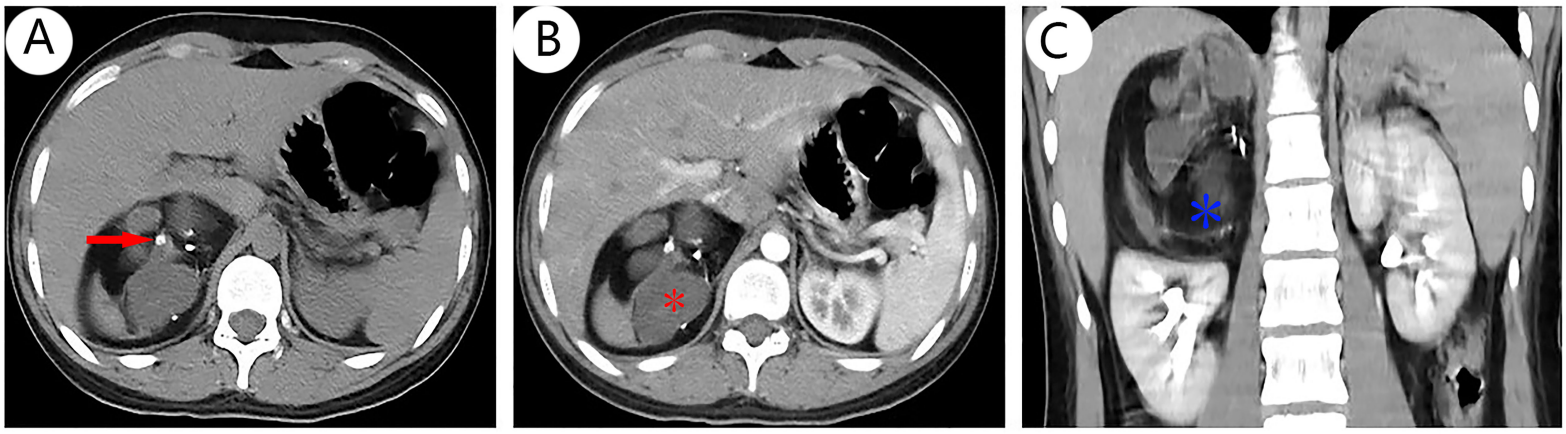
CT scan + contrast-enhanced ultrasound + 3D of adrenal gland revealed a spherical mass arising from the right adrenal gland with mixed density that was suspected to malignant tumor, a solid with multiple high-density nodules [**(A)**, red arrow], some cystic-like lesions [**(B)**, red *], and fat density shadows [**(C)**, blue *], and no obvious enhancement signals **(B, C)**. Other abdominal structures were normal and there was no evidence of distant metastasis although the right kidney was slightly displaced by the mass.

**Figure 2:**
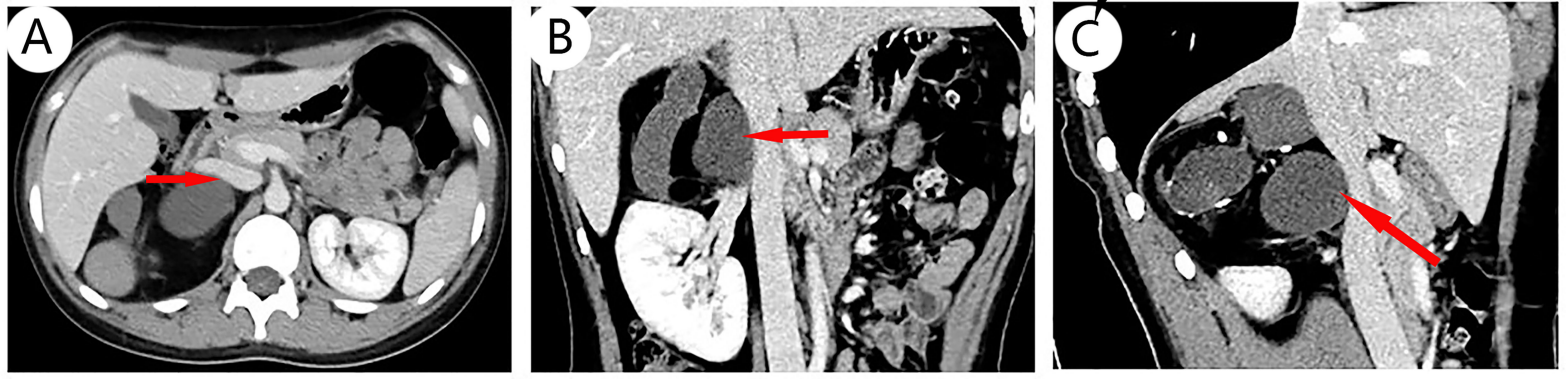
CT scan of blood vessels: No abnormalities in bilateral renal arteries were discovered in CT imaging of renal arteries. A part of inferior vena cava below the lower border of the liver segment is confined by the mass of the right adrenal gland, and the venous lumen is a local stenosis; different layers show that the vena cava is compressed, including transverse [**(A)**, arrow], coronal [**(B)**, arrow], and sagittal planes [**(C)**, arrow].

The results of chest CT scan and ECG were normal. An 88 mm × 72 mm mixed-echoic mass in the space between right kidney and liver were observed by color ultrasound of the urinary system, and CDFI showed no blood flow in the mass. CT scan + contrast-enhancement + 3D of adrenal gland showed an 88 mm × 79 mm × 69 mm mass with mixed density shadow, multiple nodular calcareous density, flake-like high density, and adipose density shadows but no obvious enhancement signals were monitored. Besides, the upper pole of right kidney was extruded by the huge mass, and there were no enlarged lymph nodes in the local area. The tumor was suspected to be a teratoma in the right adrenal gland area (**Figures 1A–C**). No abnormalities in bilateral renal arteries were discovered in the CT image of renal arteries, and the inferior vena cava below the lower border of the liver was compressed by the mass of the right adrenal gland, resulting in localized stenosis of the vena cava (**Figures 2A–C**). Through the ultrasound examination of inferior vena cava, inferior vena cava ultrasound: the inferior vena cava was visible on the tangent plane, the normal vein wall was thick, and no tumor thrombus and embolus were not found in the lumen. In addition, the CDFI discovered continuous blood flow signal without obvious filling defect, and the blood flow velocity in the liver segment of the inferior vena cava was 74 cm/s.

**Figure 3:**
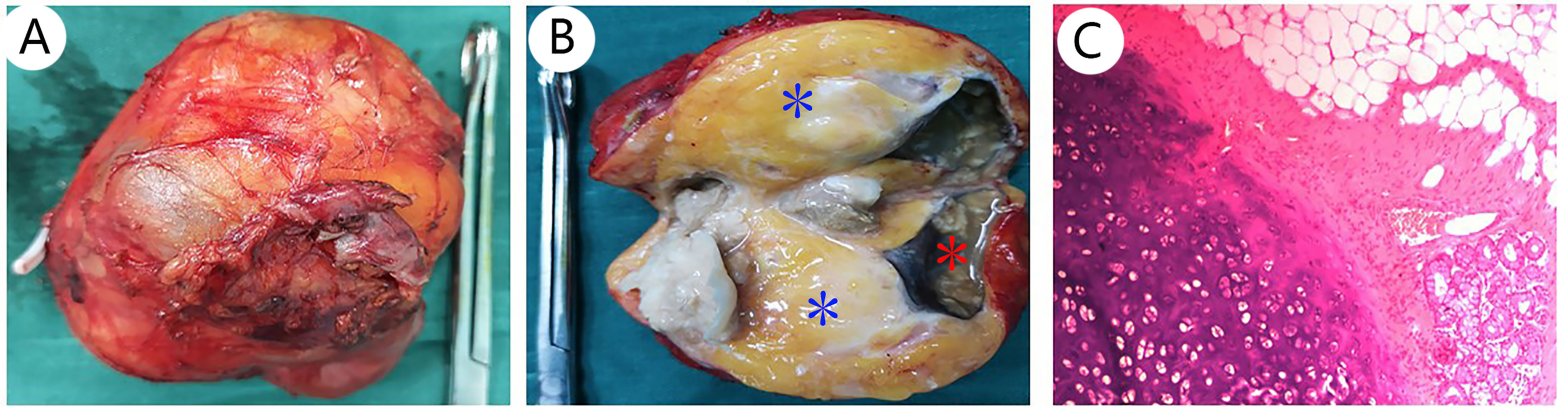
Pathological features of adrenal teratoma: **(A)** shows the gross appearance of the adrenal teratoma; the size was about 9 cm × 7 cm × 7 cm, and its envelope is intact. The cut surface is gray-yellow, namely, adipose tissue [**(B)**, blue *], cystic solid, gray necrotic fluid [**(B)**, red *], and sebum were found in part of the cyst cavity **(B)**. H&E stain, 10× **(C)**.

It is difficult for the surgeon to determine preoperatively whether the right adrenal tumor is an occult functional disease. The patient was regularly administered phenoxybenzamine 10 mg orally twice a day for 2 weeks, the blood volume was supplemented 3 days before operation, and the heart rate and blood pressure were normal. Laparoscopic right adrenal tumor resection was performed, lasting 2 h and 55 min. The size of gross tumor was about 9 cm × 7 cm × 7 cm, and its envelope was intact (**Figure 3A**). The cut surface was gray-yellow and cystic solid with gray necrotic fluid, and sebum and hair were found in part of the cyst cavity (**Figure 3B**). After the operation, the patient stopped using phenoxybenzamine tablets. Subsequently, postoperative plasma aldosterone (QKT) was 16.10 ng/dl, and cortisol (COR) was 138.3 nmol/L. Other indexes were normal. The tumor of right adrenal gland was diagnosed as a mature teratoma by pathologists (**Figure 3C**); therefore, we did not do any further genetic diagnosis. The symptoms were relieved and there were no complications when the patient was ready to leave the hospital.

## Systematic Review of Literature

PubMed databases were searched for case reports and case series of adrenal teratoma published from 1952 to 2022. The following keywords were used: (adrenal glands or adrenal gland or adrenal or glands adrenal) and (teratoma or teratoma), and 296 results were retrieved. After removing duplicates and irrelevant studies, finally, 24 publications describing 49 cases were identified (**Table 2**). In addition, primary retroperitoneal teratomas involving the adrenal glands were excluded.

**Table 2:** Review of characteristics of adrenal teratoma.

Cas No.	First author, year	Age (years) /Sex	Symptoms	Location	Size(cm)	Pathology	surgery	follow-up (months)
1	Li et al., 2011 (10)	4/F	Asymptomatic	Left	3.0×2.0×2.0	MT	OA	24
2	Li et al., 2011 (10)	38/F	Right waist pain after fatigue	Right	11.0×8.0×6.0	MT	OA	24
3	Zhao et al., 2014 (11)	21/F	Backache on the right side	Right	6.0	MT	OA	80
4	Zhao et al., 2014 (11)	35/F	Asymptomatic	Right	8.0	MT	OA	57
5	He et al., 2020 (12)	17/F	Asymptomatic	Right	7.0×2.5×2.0	MT	LA	12
6	Ban et al., 2019 (13)	60/M	left flank pain	Left	12.0×11.0×11.0	MT	NA	NA
7	Lam et al., 1999 (14)	18/F	Back pain	Left	11.0×8.0×7.0	MT	NA	84
8	Lam et al., 1999 (14)	17/M	Back pain	Right	7.5×6.0×3.0	MT	NA	6
9	Lam et al., 1999 (14)	37/F	Back pain	Left	10.0	MT	NA	96
10	Narla et al., 2016 (15)	2.5/F	right upper quadrant abdominal pain	Right	6.0×5.0×3.0	MT with carcinoid tumor	NA	8
11	Haddad et al., 2020 (16)	1/M	Asymptomatic	Left	12.0×10.9×8.5	MT	NA	6
12	Li et al., 2015 (17)	21/F	Asymptomatic	Right	8.5	MT	LA	NA
13	Li et al., 2015 (17)	16/M	Asymptomatic	Right	9.0	MT	LA	NA
14	Li et al., 2015 (17)	43/F	Asymptomatic	Left	4.9	MT	LA	NA
15	Li et al., 2015 (17)	49/F	Asymptomatic	Left	5.3	MT	LA	NA
16	Li et al., 2015 (17)	51/F	Asymptomatic	Right	2.4	MT	LA	NA
17	Zhou et al. 2018 (18)	69/F	Asymptomatic	Left	10.0×6.0×4.0	MT	NA	12
18	Zhou et al., 2018 (18)	29/F	Asymptomatic	Left	2.5×2.1×0.5	MT	LA	12
19	Ramakant et al., 2017 (19)	25F	right hypochondriac pain	Right	19.0×15.0	MT	OA	NA
20	Ersoz et al., 2011 (20)	8/M	right side pain during exercise	Right	10.0×8.0×6.0	neurocytoma arising in MT	OA	6
21	Niu et al., 2017 (21)	36/F	Asymptomatic	Right	9.0×6.0×7.5	malignant transformation of MT	LA	NA
22	McMILLAN et al., 1987 (22)	17/M	left loin pain	Left	8.0	undifferentiated MT	NA	NA
23	Wang et al., 2019 (23)	22/F	Asymptomatic	both	left:10×10.8×12.9; right:10.3×12.3×12.1	MT	LA	10
24	Castillo et al., 2006 (24)	8/M	lumbar pain after a fall	Right	8.0	MT	LA	36
25	Castillo et al., 2006 (24)	61/F	Asymptomatic	Left	8.0	MT	LA	12
26	Li et al., 2015 (25)	49/M	Asymptomatic	Right	6.0×7.0×11.0	MT	LA	8
27	Ciftci et al., 2013 (26)	0.25/M	Asymptomatic	Left	8.3×8.0	MT	NA	NA
28	Pandit et al., 2018 (6)	16/M	Asymptomatic	Left	12.0×10.0	MT	OA	12
29	Zhong et al., 2019 (27)	59/F	Flank pain	Left	10.6	MT	OA	124
30	Zhong et al., 2019 (27)	54/F	Asymptomatic	Right	10.2	MT	OA	124
31	Zhong et al., 2019 (27)	22/F	Abdominal pain	Right	10.0	MT	OA	109
32	Zhong et al., 2019 (27)	48/F	Asymptomatic	Right	9.6	MT	LA	101
33	Zhong et al., 2019 (27)	26/M	Flank pain	Right	4.0	MT	LA	90
34	Zhong et al., 2019 (27)	18/F	Abdominal pain	Right	8.8	MT	LA	88
35	Zhong et al., 2019 (27)	55/F	Asymptomatic	Left	14.0	MT	OA	69
36	Zhong et al., 2019 (27)	28/F	Asymptomatic	Right	4.5	MT	LA	60
37	Zhong et al., 2019 (27)	29/F	Asymptomatic	Right	7.8	MT	LA	56
38	Zhong et al., 2019 (27)	29/F	Asymptomatic	Left	7.0	MT	LA	56
39	Zhong et al., 2019 (27)	72/F	Asymptomatic	Right	6.0	MT	OA	55
40	Zhong et al., 2019 (27)	28/F	Asymptomatic	Left	9.5	MT	OA	76
41	Zhong et al., 2019 (27)	41/F	Asymptomatic	Left	18.0	MT	OA	43
42	Zhong et al., 2019 (27)	45/M	Asymptomatic	Left	6.8	MT	LA	28
43	Bhatia et al., 2016 (28)	24/F	left hypochondrium pain	Left	7.6×6.5	MT	NA	NA
44	Kuo et al., 2017 (29)	26/M	right upper quadrant and right flank pain	Right	8.5×5.5×4.8	MT	OA	NA
45	Kuo et al., 2017 (29)	29/F	Asymptomatic	Left	2.5×2.1×0.5	MT	LA	NA
46	Kuo et al., 2017 (29)	24/F	left lower quadrant abdominal pain	Left	11.5×9.0×3.0	MT	LA	NA
47	Garg et al., 2017 (30)	0.25/F	Asymptomatic	Right	10.0×10.0×8.0	MT	OA	NA
48	Tang et al., 2014 (31)	39/F	dizzy	Right	22.5×17.0×7.0	MT	NA	18
49	Nadeem et al., 2015 (32)	19/M	vague pain in right flank	Right	8.0×6.0×4.0	MT	OA	12

F, female; M, male; MT, mature teratoma; OA, open adrenalectomy; LA, laparoscopic adrenalectomy; NA, not available.

The series of review comprised 49 patients (14 men and 35 women) with a mean age of 30.3 years. Adrenal-related hormones were normal in all patients and 27 (55%) patients were clinically asymptomatic. The majority of adrenal teratomas were unilateral, with only one case of bilateral adrenal involvement. In contrast to previous reports, the current data suggest that right-sided adrenal teratomas are more common. In most cases, mature fat and calcified tissue were observed. All cases were surgically resected and histopathologically confirmed as teratomas. Most of the adrenal teratomas were mature teratomas, two of which were within focal carcinomas and neuroblastomas, respectively, and one was a malignant transformation of a teratoma into an adenocarcinoma. Only one case was an undifferentiated malignant teratoma. Thirty-four of the 49 patients were followed up for 3 months to 10 years, and all had a good prognosis with no tumor recurrence or other discomfort during the follow-up period.

## Discussion

The teratoma is a rare neoplasm; according to different original sites, teratomas can be categorized into gonadal and extragonadal teratomas, and retroperitoneal space is a site of the extragonadal location. When primary retroperitoneal neoplasms in adults were found that represent an infrequent mass (4), the primary adrenal teratomas are extremely rare. Previous reports demonstrated that adrenal teratomas tend to occur more often in women than in men and involve predominantly the left adrenal gland (29), which is consistent with the results of our systematic review in terms of gender, but the reviews suggest that teratomas occur mainly in the right adrenal gland. However, Zhong et al. found an almost identical distribution on both sides (33). In this case, it occurred in the right adrenal gland in a female patient.

Primary adrenal teratoma has no distinctive clinical presentation with normal adrenal-related hormones, presenting as “incidentalomas”, and they are not clearly distinguishable from other adrenal tumor with no endocrine function. In clinical practices, primary adrenal teratoma is usually found in medical examination and diagnosis of other functional adrenal tumors, such as adrenal cortical adenoma or pheochromocytoma (34). Therefore, adrenal teratomas should be differentiated from adrenal incidentalomas, non-functioning adrenal lesions and adrenal cystic lesions (35). Adrenal teratomas are usually large because there is enough retroperitoneal space for its growth. However, as the tumor grows, about half of the patients may suffer from abdominal distension, abdominal pain, back pain, or even intestinal obstruction due to the tumor pressing on the surrounding organs and blood vessels (36). A previous data indicated that the median primary adrenal teratomas mean diameter was 8.25 cm (37). In this case, the complaint of the patient was intermittent pain in the right waist and abdomen and the tumor size was 9 cm × 7 cm × 7 cm (**Figure 1**).

Adrenal teratomas are usually asymptomatic or have non-specific complaints, and laboratory tests are generally normal, thus posing a significant preoperative diagnostic challenge and relying primarily on imaging for diagnosis. Ultrasound usually shows a heterogeneously echogenic mass including hyperechoic calcifications and ossifications, as well as hypoechoic fatty and cystic areas (38). CT indicates a mixed density lesion with hypointense cystic and fatty areas and hyperintense calcifications (39). MRI presents as mixed signals on both T1- and T2-weighted images, with a high fat content. In addition, CT and MRI can also clarify the location of tumor growth and its relationship to surrounding organs, facilitating the assessment of surgical approaches and risks. However, other adrenal tumors with a fatty component are considered for differentiation from adrenal teratomas, including myelolipoma, lipoma, angiomyolipoma, and liposarcoma (12). In previously reported cases, a number of adrenal teratomas have been misdiagnosed preoperatively. Therefore, postoperative pathological examination is usually required to confirm the diagnosis. In this case, a mixed-echoic mass near the adrenal gland were observed by color ultrasound, and CT showed an 88 mm × 79 mm × 69 mm mass with mixed density shadow, multiple nodular calcareous density, flake-like high density, and adipose density shadows, which is consistent with the imaging features of adrenal teratoma. The patient was diagnosed preoperatively with a right adrenal teratoma.

Most mature teratomas are benign tumors, and the surrounding organs can be compressed by the growing tumor, even with the possibility of tumor rupture, bleeding, and infection. Once diagnosed, surgery should be performed as soon as possible. Laparoscopic surgery has become the “gold standard” for the treatment of adrenal tumors smaller than 6 cm (40). For adrenal tumors larger than 6 cm, open surgery is the main approach to resection of the tumor. However, scholars have pointed out that laparoscopic surgery is safe and feasible for benign adrenal tumors, as long as there is no local invasion on preoperative imaging or intraoperatively (23), and laparoscopic treatment of adrenal tumors larger than 6 cm has become feasible (29). In our systematic review, at least 12 patients with a tumor larger than 6 cm in diameter were successfully resected by laparoscopic adrenalectomy without complications, which further confirms the feasibility of this view. In this case, it is difficult to determine whether the right adrenal tumor is an occult functional disease. The patient was regularly administered phenoxybenzamine 10 mg orally twice a day for 2 weeks, and the blood volume was supplemented 3 days before operation, with these being the strengths of our case, the heart rate and blood pressure were normal. Because a part of the inferior vena cava below the lower border of the liver segment is confined by the mass of the right adrenal gland, the venous lumen is a local stenosis. After general anesthesia, transabdominal right adrenal tumor resection under the modified left lateral position was performed with no complications.

The etiology of teratomas is not known and the pathological classification includes mature teratomas and immature teratomas, and the pathological criteria for benign lesions include the following four aspects: (I) absence of malignant or immature components in the tumor, (II) absence of other similar lesions elsewhere in the body, (III) normal levels of AFP and HCG, and (IV) absence of recurrence at follow-up (17). Although the prognosis of mature teratoma is good, it has been reported that about 1.46% of mature teratomas will have malignant potential (13). It is necessary to regularly follow up, and radiotherapy and chemotherapy are not necessary. For immature teratoma, the rate of recurrence and metastasis is high, and radiotherapy and chemotherapy are still required postoperatively with a lifelong follow-up (9). For the cases in this report, resected tumors were identified as mature cystic teratomas by pathologists (**Figure 3C**). As the cases were recent, there was not enough time for follow-up, which was a disadvantage in this case. However, our reviews showed that 34 cases had no recurrence or other discomfort at the 6-month to 10-year follow-up.

Elevated AFP, β-HCG, and CEA were found in some immature teratomas, which were of great significance for preoperative diagnosis and prognosis (41). For the immature adrenal teratomas, chemotherapy should be supplemented after surgery, but there is currently no standard chemotherapy regimen. BEP chemotherapy regimen (bleomycin, etoposide, cisplatin) was used for a patient with immature adrenal teratomas in a review, and no recurrence or metastasis was found after 13 months of follow-up (13). At present, there is no sensitive indicators for monitoring relapse of adrenal teratoma. The level of AFP was correlated with the recurrence of teratoma, and it can be used as a predictive index for curative effect (42). The patient in this report is a mature teratoma; now, we do not know the long-term prognosis for only 1 month post-operation. Strict regular follow-up is needed, and it is necessary to detect AFP and adrenal CT in the future.

## Patient Perspective

My intermittent pain in my right lower back and abdomen was affecting my daily life and work, causing me great distress. After the doctor’s consultation, he helped me to make a correct diagnosis and opted for minimally invasive laparoscopic surgery to completely remove the tumor from my body and eliminate the pain in my upper right abdomen. My fears and worries about the tumor disappeared, especially after the pathology confirmed the diagnosis of a mature teratoma. I was able to achieve both physical and psychological healing. I consider that I have received a very successful treatment. I agreed to share my medical history and signed an informed consent form.

## Conclusion

We have described the features of imaging, pathology, and the treatment of the typical primary adrenal teratoma to further raise people’ awareness of this rare disease, and primary adrenal teratomas should be included in the differential diagnosis of adrenal masses.

## Data Availability Statement

The raw data supporting the conclusions of this article will be made available by the authors, without undue reservation.

## Ethics Statement

The studies involving human participants were reviewed and approved by the Ethics Committee of the Affiliated Hospital of Zunyi Medical University. The patients/participants provided their written informed consent to participate in this study. Written informed consent was obtained from the individual(s) for the publication of any potentially identifiable images or data included in this article.

## Author Contributions

XW and XGL were the patient’s urologists, reviewed the literature, and contributed to manuscript drafting. HC, WX, XH and PS were responsible for the revision of the manuscript for important intellectual content. All authors contributed to the article and approved the submitted version.

## Funding

This study was supported by the National Natural Science Foundation of China (grant no. 81860524) and grants from the Department of Science and Technology of Guizhou Province (grant no. 386, in 2021-year).

## Conflict of Interest

The authors declare that the research was conducted in the absence of any commercial or financial relationships that could be construed as a potential conflict of interest.

## Publisher’s Note

All claims expressed in this article are solely those of the authors and do not necessarily represent those of their affiliated organizations, or those of the publisher, the editors and the reviewers. Any product that may be evaluated in this article, or claim that may be made by its manufacturer, is not guaranteed or endorsed by the publisher.

## Abbreviations

AFP, Alpha-fetoprotein; CT, Computed tomography; CA19-9, Carbohydrate antigen 19-9; CA15-3, Carbohydrate antigen 15-3; CA125, Carbohydrate antigen 125; CDFI, Color Doppler flow image; CEA, Carcinoembryonic antigen; ECG, Electrocardiograph; β-HCG, Human chorionic gonadotrophin; MRI, Magnetic resonance imaging.
